# Host plant forensics and olfactory-based detection in Afro-tropical mosquito disease vectors

**DOI:** 10.1371/journal.pntd.0006185

**Published:** 2018-02-20

**Authors:** Vincent O. Nyasembe, David P. Tchouassi, Christian W. W. Pirk, Catherine L. Sole, Baldwyn Torto

**Affiliations:** 1 International Centre of Insect Physiology and Ecology, Nairobi, Kenya; 2 Department of Zoology and Entomology, University of Pretoria, Hatfield, South Africa; Yale School of Public Health, UNITED STATES

## Abstract

The global spread of vector-borne diseases remains a worrying public health threat, raising the need for development of new combat strategies for vector control. Knowledge of vector ecology can be exploited in this regard, including plant feeding; a critical resource that mosquitoes of both sexes rely on for survival and other metabolic processes. However, the identity of plant species mosquitoes feed on in nature remains largely unknown. By testing the hypothesis about selectivity in plant feeding, we employed a DNA-based approach targeting trnH-psbA and matK genes and identified host plants of field-collected Afro-tropical mosquito vectors of dengue, Rift Valley fever and malaria being among the most important mosquito-borne diseases in East Africa. These included three plant species for *Aedes aegypti* (dengue), two for both *Aedes mcintoshi* and *Aedes ochraceus* (Rift Valley fever) and five for *Anopheles gambiae* (malaria). Since plant feeding is mediated by olfactory cues, we further sought to identify specific odor signatures that may modulate host plant location. Using coupled gas chromatography (GC)-electroantennographic detection, GC/mass spectrometry and electroantennogram analyses, we identified a total of 21 antennally-active components variably detected by *Ae*. *aegypti*, *Ae*. *mcintoshi* and *An*. *gambiae* from their respective host plants. Whereas *Ae*. *aegypti* predominantly detected benzenoids, *Ae*. *mcintoshi* detected mainly aldehydes while *An*. *gambiae* detected sesquiterpenes and alkenes. Interestingly, the monoterpenes β-myrcene and (*E*)-β-ocimene were consistently detected by all the mosquito species and present in all the identified host plants, suggesting that they may serve as signature cues in plant location. This study highlights the utility of molecular approaches in identifying specific vector-plant associations, which can be exploited in maximizing control strategies such as such as attractive toxic sugar bait and odor-bait technology.

## Author summary

Plants play an important role in the fitness of mosquito disease vectors, yet the identity of plant species that they feed on in their natural habitats remains largely unknown. In this study, we employed DNA barcoding to identify the plant species fed upon by *Aedes aegypti*, *Aedes mcintoshi*, *Aedes ochraceus* and *Anopheles gambiae* in their natural habitats. Since plant feeding is mediated by olfactory cues, with potential application as attractant-based tools for vector surveillance, we identified specific odor signatures that may modulate host plant location. Our findings showed preference in odor detection among the vectors for different compound classes; benzenoids for *Ae*. *aegypti*, aldehydes for *Ae*. *mcintoshi* and sesquiterpenes and alkenes for *An*. *gambiae*. This study highlights the utility of molecular approaches in identifying specific vector-plant associations, a knowledge which can be exploited in maximizing vector control strategies such as attractive toxic sugar bait. Furthermore, the elucidation of potential odor signature lays foundation for development of plant odor-bait technology which is critical for surveillance of different mosquito disease vectors of varying physiological states and the pathogens they transmit.

## Introduction

There has been an increase in the incidence of vector-borne diseases, key among them arboviral diseases such as dengue, chikungunya, Rift Valley fever (RVF) and zika. While dengue predominantly affects Asian countries, increasing outbreaks in East African coastal regions have become evident in recent times [1–3]. Rift Valley fever which mainly occurs in Africa, with foci in East Africa, is rapidly spreading eastwards into Asia and the Arabian Peninsula [4–6]. The recent upsurge in dengue incidence has been attributed to rapid and unplanned urbanization which creates conducive breeding habitats for the key mosquito vector of the disease, *Aedes aegypti* [3, 7]. On the other hand, RVF is an epizootic disease mainly associated with devastating outbreaks following widespread elevated rainfall, leading to flooding that creates favorable breeding sites for the primary mosquito vectors *Aedes mcintoshi* and *Aedes ochraceus* [5, 8]. In East Africa, in particular Kenya, the public health burden due to these arboviral diseases is further compounded by the endemicity of the parasitic malaria disease transmitted by certain species of the *Anopheles* mosquito [9]. Globally, vector-borne diseases pose risk of infection to more than half of the world population with more than a million deaths annually [10]. Consequently, there is renewed effort to come up with new disease control measures and vector control forms a key pillar in these efforts. Detailed understanding of the vector ecology is needed in search for novel control strategies.

Plant feeding plays a critical role in the bio-ecology of mosquito disease vectors. Several studies have demonstrated that both sexes of different mosquito species forage on plants to obtain carbohydrates required for metabolic processes vital for their survival [11–13]. Besides providing a ready source of energy for flight, fecundity and cell metabolism [12, 14, 15], plant carbohydrates are also utilized during diapause by mosquitoes such as *Culex pipiens* to synthesise lipid reserves [16–18]. The availability of host plants has also been shown to extend survival of *Anopheles gambiae* and *An*. *sergentii*, likely allowing for the completion of sporogonic cycle of malaria parasites and thereby increasing disease transmission potential of these vectors [19–22]. On the other hand, abundance of flowering plants has been linked to reduced human biting behavior by mosquito disease vectors, which can impact either positively or negatively on disease transmission potential depending on the infection status of the mosquitoes [13, 20, 23, 24]. In addition, studies have shown that both mosquitoes and sandflies imbibe plant secondary metabolites during plant feeding, some of which reduce parasite load in the vector [21, 25, 26]. This has led to the hypothesis of possible self medication by these disease vectors [15, 26] opening up new avenues for exploiting phytochemicals in development of novel chemotherapeutics against the pathogens they transmit. Thus, beyond the provision of nutrients, understanding plant feeding in disease vectors offers promising opportunities for development of new control strategies against the myriad of vector borne diseases. Despite this, little is known about plant feeding behavior of mosquito vectors of dengue and RVF.

Previous studies have demonstrated that mosquitoes are highly selective in their choice of plants [27–30]. These inferences were drawn from semi-field and field experiments which either involved feeding mosquitoes on randomly selected peri-domestic plants to determine their acceptability [27, 28] or determining the attraction of mosquitoes to randomly selected fruits/seedpods and flowering plants [29–31]. Determination of plant feeding among various mosquito species has mainly been based on analytical techniques such as cold anthrone tests to detect fructose in the crop of field collected mosquitoes, chromatographic methods to detect plant sugars and cellulose staining to detect plant tissue feeding [12, 16, 19, 23, 24, 28, 32], with little to no direct field observations of mosquitoes feeding on plants [33, 34]. While contributing immensely towards our understanding of the role of plant feeding in the vectorial capacity of mosquitoes, these methods are, however, limited by their inadequacy in determining the precise host plants in the natural mosquito habitats. As diverse plants often occur in each habitat, the critical question of which plants, if any, are foraged upon by mosquito disease vectors, remains unanswered.

Recent advances have seen the application of DNA barcoding targeting specific genes to profile plant species fed upon by disease vectors [35, 36]. However, this has not been applied for any Afro-tropical disease vector, thus far. By employing DNA barcoding targeting multiple gene loci, we tested the hypothesis that Afro-tropical disease vectors feed on certain plants in their respective ecologies. We focused on four mosquito species which transmit dengue (*Aedes aegypti*), Rift Valley fever (*Aedes mcintoshi* and *Aedes ochraceus*) and malaria (*An*. *gambiae*) [3, 37–39]. These diseases rank among the most important vector-borne diseases in Kenya with some having been associated with large outbreaks affecting humans in the recent past [2, 5, 6, 9]. Given the central role of olfactory cues in locating this key plant resource [40–42], we further used coupled gas chromatography/mass spectrometry and electrophysiological assays to test the hypothesis that these disease vectors use unique odor bouquet to locate their suitable natural host plant. Our results show that the four Afro-tropical mosquito species feed on certain plant species within their ecological range and detect common and specific chemical cues to locate their suitable host plants. This study provides useful insight that can inform vector control strategies targeting plant feeding behavior such as attractive toxic sugar bait and odor bait technology.

## Matherials and methods

### Mosquito sample collection

Mosquito samples were obtained from three sites in Kenya: *Ae*. *aegypti* from Kilifi (3.6333° S and 39.8500° E) in the coastal region with high dengue endemicity [2, 3], *Ae*. *mcintoshi* and *Ae*. *ochraceus* from Ijara (1.5988° S and 40.5135° E) in north eastern Kenya which is a Rift Valley fever endemic region [5, 6] and *An*. *gambiae* s.l. from Ahero (0°10’S, 34°55’E) which is a malaria endemic area in western Kenya [9]. The trapping methods used to collect mosquito samples are described in detail in Nyasembe et al. [42]. Briefly, unlit CDC traps separately baited with linalool oxide (LO), BioGent (BG) lure and HONAD (a mixture of heptanal, octanal, nonanal and decanal) formulated from mammalian odor by Tchouassi et al. [37] were used to trap *Ae*. *mcintoshi*, *Ae*. *ochraceus* and *An*. *gambiae*; while BG sentinel traps separately baited with LO and BG lure, were used to trap *Ae*. *aegypti*. The traps were also either baited with or without carbon dioxide in the form of dry ice at all the three sites. All trappings were carried out outdoors. In Ahero and Kilifi, traps were placed in three distinct settings i) close to the homestead, ii) close to breeding sites *a priori* identified as positive for the specific mosquito larvae, and iii) in vegetation away from human dwelling [3, 39, 43, 44]. In Ijara, the traps were set at two distinct settings i) next to ‘dambos’ which serve as breeding sites for *Ae*. *mcintoshi* and *Ae*. *ochraceus* and ii) in bushy grasslands where pastoralists graze their livestock [6, 37, 38]. In Kilifi, trappings were carried out both during the day and night (informed by the diurnal nature of *Ae*. *aegypti*) while in Ahero and Ijara trappings were carried out during the night only (due to the nocturnal nature of mosquito vectors in these localities). Traps were emptied after 12 h and the collected mosquitoes immobilized by placing on dry ice, immediately frozen in liquid nitrogen and transported to *icipe* laboratories in Nairobi for further processing.

### Sample preparation

To prepare the samples for biochemical and molecular analyses, individual mosquitoes were submerged in a solution of 0.5% hypochlorite, agitated gently for 1 min with forceps, and then rinsed in double distilled water (ddH_2_0) for 1 min. This was to remove any plant debris that may have been on the outside of the insect, which could otherwise contaminate the sample. The mosquitoes were then placed individually in a 1.5 ml sterile Eppendorf tube and macerated using round-tipped glass rods sterilized through the flame of a Bunsen burner. One hundred microlitres of absolute ethanol was added and the solution homogenized. Two sets of controls were used as follows: a) laboratory-reared *An*. *gambiae* s.s. fed on *Parthenium hysterophorus* (Asteraceae) overnight, and b) *An*. *gambiae* aspirated directly from *P*. *hysterophorus* field in Ahero using a backpack aspirator (3” IN-LINE BLOWER, John W. Hock Company, Gainesville, FL, USA). All mosquitoes from the controls were prepared as described above.

### Determination of evidence of recent plant feeding in field collected mosquitoes

This was done using the cold anthrone test as described by van Handel et al., [45] as a quick initial test to detect fructose. Aliquots (50 μl) of the prepared mosquito homogenate were individually placed in the wells of a flat bottomed 96-well microtiter plate followed by 300 μl of the reaction solution comprising 0.15% anthrone (Sigma) (wt/vol) in 71.7% sulphuric acid. This was incubated at 25 ^o^C for 60 min before being examined for color changes. In the presence of fructose, the reaction mixture changed its color from yellow to blue. The remaining aliquot of fructose-positive samples was subjected to plant DNA extraction as described below.

### Extraction of plant DNA from mosquitoes

Plant DNA was extracted from the homogenized samples of fructose-positive mosquitoes only using the manufacturer’s protocol described by DNeasy Plant Minikit- (QIAGEN, USA) with a minor modification. The incubation period with lysis buffer AP1 and RNase was extended by 30 min while that with the elution Buffer AE was extended for 3 hr. The extracted DNA was stored at -20°C until use in PCR amplification.

### PCR amplification and sequencing

Plant DNA extracted from the fructose-positive mosquito specimens was amplified targeting the trnH-psbA intergenic spacer region and maturase K (matK) gene (Table 1) using established primers. The use of more than one target was to maximize on the detection possibility as individual genes selectively amplify certain plant families [46]. Each PCR reaction (carried out in a volume of 20 μl) consisted of 7 μl template DNA, 10 μl 2x HotStarTaq Master Mix (HotStarTaq Plus Master Mix Kit, Qiagen), 0.5 μM of each primer, and 2 μl of RNase free water. A PCR negative control (RNase-free water) was routinely used. Samples were amplified using Veriti 96-well Thermal Cycler (Singapore). For trnH-psbA, the cycling parameters were 94 ^o^C for 1 min, followed by 45 cycles of 94 ^o^C for 1 min, 55 ^o^C for 40 sec and 72 ^o^C for 1 min, and final extension at 72 ^o^C for 10 min. Similar cycling conditions were used for matK amplification with the annealing temperature set at 48 ^o^C.

**Table 1:** Forward and reverse primer sequences for three gene targets used to identify natural host plants of dengue, Rift Valley fever and malaria mosquito disease vectors.

Primer	Direction	Sequence (5’-3’)	Reference
trnH-psbA	trnH	CGCGCATGGTGGATTCACAATCC	Shaw et al., 2005
	psbA	GTTATGCATGAACGTAATGCT	Shaw et al., 2005
matK	2.1 forward	CCTATCCATCTGGAAATCTTAG	Kress et al., 2005
	5 reverse	GTTCTAGCACAAGAAAGTCG	Kress et al., 2005

Successful amplifications were confirmed by visualizing PCR amplicons in 1% agarose gel electrophoresis. They were purified using the Exo/SAP-IT Kit for PCR product (Affymetrix Inc., USA) as per the manufacturer’s instructions and outsourced for bidirectional sequencing to Inqaba Biotechnological Industries (Pty) Ltd (Pretoria, South Africa).

The obtained plant DNA sequences for each gene were cleaned, edited and compared to reference sequences in the GenBank database [47]. In GenBank, the ‘megablast’ search option of nucleotide Basic Local Alignment Search Tool (BLASTn) [48] algorithm was used with the default search parameters. The hits with sequence identity above 96% were retrieved and added to the original sample query sequences. The sequences were aligned using ClustalW in MEGA 6 [49]. Aligned matrices were used to construct p-distance phylogenetic tree using the Neighbor Joining method for individual genes with 1000 bootstraps. Nodal support was evaluated by bootstrapping with values of 95% or more considered significant.

### Confirmation of identified mosquito host plants

Further steps to confirm the plant identity included on-site identification within the specific ecologies from where the mosquitoes were sampled by a plant taxonomist (Simon Mathenge, retired from the Herbarium, Department of Botany, University of Nairobi) and comparison to established botanical database by the National Museum of Kenya (http://www.museums.or.ke).

Leaves and flowers (where applicable) of identified plants were sampled from the respective field sites for DNA extraction and sequencing. The samples were cleaned using double distilled water before obtaining approximately 100 mg wet weight of the sample which were placed in sterile 1.5 ml Eppendorf tubes. The plant samples were homogenized and DNA extracted using the DNeasy Plant Mini Kit as described above. The obtained plant DNA was similarly amplified for trnH-psbA and matK genes, processed and sent for sequencing as described above. The sequences were then aligned with those from the mosquitoes and phylogenetic trees obtained. Nodal support was evaluated by bootstrapping with values of 95% or more considered significant.

The haplotypes generated from this study have been deposited in GenBank under accession numbers MG573108, MG573126 –MG573131 (RVF vectors host plant trnH-psbA gene sequences), MG573132 –MG573139 (dengue vector host plant trnH-psbA gene sequences), MG573109 –MG573125 (malaria vector host plant trnH-psbA gene sequences), and KY308115—KY398121 (malaria vector host plant matK gene sequences).

### Collection of headspace volatiles from the identified natural mosquito host plants

Headspace VOCs were collected from five of the confirmed natural host plants for *Ae*. *aegypti*, *Ae*. *mcintoshi*, *Ae*. *ochraceus* and *An*. *gambiae*. The five plants included *Pithecellobium dulce* (Fabaceae), *Opuntia ficus-indica* (Cactaceae), *Leonotis nepetifolia* (Lamiaceae), *Senna alata* (Fabaceae) and *Ricinus communis* (Euphorbiaceae). This was done by collecting the headspace volatiles from these plants *in situ* at their natural habitats using a portable field pump (Analytical Research Systems, Gainesville, Florida, USA). The aerial parts of an intact plant were gently enclosed in an air-tight oven bag (Reynolds, Richmond, VA, USA) and charcoal filtered air passed over the plant at a flow rate of 350 ml/min into a Super-Q adsorbent trap (30 mg, Analytical Research Systems, Gainesville, Florida, USA). The aerial plant parts enclosed in the oven bags included leaves, flowers and pods of *P*. *dulce* and *S*. *alata*, leaves and flowers of *L*. *nepetifolia*, leaves and leaf stalks of *R*. *communis*, and leaves, flowers and fruits of *O*. *ficus-indica*. For all plant species, volatiles were collected for 12 hr during the day and 12 hr at night and replicated three times using different plants in each replicate. The Super-Q traps were eluted with 200 μl GC/GC-MS-grade dichloromethane (DCM) (Burdick and Jackson, Muskegon, Michigan, USA) and the eluents stored at -80 °C until analysis.

### Analysis of volatiles

For quantification and identification of the constituent compounds of the plant volatiles, an aliquot (1 μl) of each sample was injected into a gas chromatograph (Agilent technologies-7890) coupled to inert XL EI/CI mass spectrophotometer (5975C, EI, 70eV, Agilent, Palo Alto, Califonia, USA) (GC/MS) in a splitless injection mode. The GC was equipped with an HP-5 column (30 m x 0.25 mm ID x 0.25 μm film thickness, Agilent, Palo Alto, California, USA), with helium as the carrier gas at a flow rate of 1.2 ml/min. The oven temperature was held at 35 °C for 5 min, then programmed to increase at 10 °C/min to 280 °C and maintained at this temperature for 10 min. The volatile organic compounds were identified by comparing their mass spectra with library data (Adams2.L, Chemecol.L and NIST05a.L) and with those of authentic standards where possible (see sources and purity under chemical section below). The absolute areas of each constituent as calculated by the NIST05a.L software was used to estimate their amounts using an external calibration equation generated from known amounts of authentic compounds.

### Electrophysiological assays

To isolate the specific VOCs that are detected by the different mosquito disease vectors and their preferred natural host plants, wild caught adult *Ae*. *aegypti*, *Ae*. *mcintoshi* and *An*. *gambiae* s.l., were collected from their respective habitats using methods described above and transported alive to the *icipe* laboratories in Nairobi under high containment level and directly used in electrophysiological assays. The trapped mosquitoes were aspirated into 30 x 30 x 30 cm cages and provided with 10% glucose solution soaked in cotton wool during transportation. The tops of the cages were covered with a moist towel to maintain high humidity. Once at *icipe*, the mosquitoes were kept in a high containment animal rearing unit at a temperature of 27–31°C and average humidity of 80%. Only female mosquitoes were used for electrophysiological assays and they were starved for 2 hr before experimentation. *Anopheles gambiae* s.s. antennal responses to *R*. *communis* headspace volatiles had been tested in our previous study [40], hence was not repeated in this study. In addition, *Ae*. *ochraceus* was not used in these studies as none were collected during this field sampling.

Coupled gas chromatography/electro-antennographic detection (GC/EAD) analyses were performed as described by Nyasembe et al. [40]. Briefly, 5 μl of volatile samples were analyzed using a Hewlett-Packard (HP) 5890 Series II gas chromatograph equipped with an HP-5 column (30 m x 0.25 mm ID x. 0.25 μm film thickness, Agilent, Palo Alto, California, USA) with nitrogen as the carrier gas at 1 ml/min. Volatiles were analyzed in the splitless mode at an injector temperature of 280 °C and a split valve delay of 5 min. The oven temperature was held at 35°C for 3 min, then programmed at 10 °C/min to 280 °C and maintained at this temperature for 10 min. The column effluent was split 1:1 after addition of make-up nitrogen gas for simultaneous detection by flame ionization detector (FID) and EAD. For EAD detection, silver-coated wires in drawn-out glass capillaries (1.5 mm I.D.) filled with Ringer saline solution served as reference and recording electrodes. Live mounting in which the mosquito was restrained with an adhesive tape with the reference electrode connected to the base of the head and the recording electrode connected to the tip of the antennae. The analogue signal was detected through a probe (INR-II, Syntech, Hilversum, the Netherlands), captured and processed with an intelligent data acquisition controller (IDAC-4, Syntech, the Netherlands), and later analyzed with EAG 2000, software (Syntech). FID signals from the respective host plant volatiles that elicited repeated antennal responses in at least three replicates using fresh antennae were designated as EAD-active compounds and identified by matching them with corresponding GC/MS data and those of authentic standards.

EAG puffs were used to confirm the detection of seven EAD-active components which elicited antennal responses using synthetic standards. The seven compounds were selected based on either being detected by more than one mosquito species from the volatiles from their respective host plants, or by the same mosquito species from volatiles of different host plants. The synthetic standards were prepared at a concentration of 1 ng/μl, 2 ng/μl and 4 ng/μl in dichloromethane (Sigma Aldrich, 99.9%) and separately delivered as puffs on 1 cm X 1 cm filter paper placed in Pasteur pipettes. The puffs were delivered at 1 min interval, allowing the antennae to equilibrate post-exposure. To correct for variability in response, responses to blanks (filter paper laced with solvent only) were subtracted from each sample and antennal response values were normalized to a standard stimulus set at 100% (2 ng/μl 1-octen-3-ol, chosen based on its known attractiveness to hematophagous insects [41]. EAG puffs were replicated nine times for each dose of every stimulus.

### Chemicals used

The synthetic standards of the following EAD-active compounds were used: hexanal (Sigma Aldrich, 99%), (*E*)-2-hexenol (Aldrich, 96%), benzaldehyde (Sigma Aldrich, 99.5), β-myrcene (Sigma Aldrich, 99%), ocimene (International Flavors and Fragrance, New York, USA, (Z)-β-ocimene = 27%, (E)-β-ocimene = 67% and allo-ocimene = 6%), linalool oxide (Sigma Aldrich, mixture of stereoisomers with furanoid form, 99.5% and 0.5% pyranoid form), indole (Sigma Aldrich, 99%) and 1-octen-3-ol (Fluka Chemica, racemic mixture of R and S 98%).

### Statistical analysis

To determine if there was any significant difference in the volatile profiles of the five plant species, ten most abundant volatile constituents in each plant species were selected. Attempts were then made to retrieve each of these compounds from the VOCs analyzed for the rest of the plant species, yielding a total of 26 different compounds. The absolute areas of these compounds were then measured and converted into a percentage of the total. These percentages were then subjected to Principal Component Analysis (PCA) to determine which ones, if any, are important in explaining the variation in the odor profiles of the five different plant species. Quantitative differences in VOCs of the five different plant species were detected using Univariate analysis of variance and Tukey post hoc test. Differences between the antennal dose responses and between the three different mosquito species, were detected using ANOVA and Tukey post hoc test. All statistical analyses were carried out at 95% confidence interval using R 2.15.1 software [50].

## Results

### Evidence of plant feeding among wild-caught Afro-tropical mosquito species

By applying the cold anthrone test to detect fructose as evidence of recent plant feeding, we established the degree of plant feeding among the females of four Afro-tropical mosquito species *Aedes aegypti* (dengue vector), *Aedes mcintoshi*, *Ae*. *ochraceus* (RVF vectors) and *Anopheles gambiae* s.l. (malaria vector) trapped from different habitats in Kenya during the long rainy season (April-June, 2014). Since no male mosquitoes were collected for RVF and malaria vectors, this analysis was limited to only female mosquitoes for all the four species. We found evidence of recent plant feeding in *Ae*. *aegypti* (17%, n = 245), *Ae*. *mcintoshi* (56%, n = 68), *Ae*. *ochraceus* (65%, n = 50) and *An*. *gambiae* (24%, n = 146).

### Afro-tropical mosquito species feed on diverse plant species in their natural habitats

To determine the identities of the plant species fed upon by these mosquito species in their natural habitats, we subjected aliquots of samples that tested positive for the anthrone test to DNA extraction followed by amplification targeting two plant genes; trnH-psbA and matK; and then sequencing. We observed that the success rates in amplification of plant DNA from the mosquito crop differed significantly between the two gene targets; trnH-psbA (24.5%) and matK (8.8%) (*P* < 0.05; Table 2). Similarly, sequencing success rates differed significantly between trnH-psbA (16.4%) and matK (1.9%) (*P* < 0.05; Table 2). The sequenced fragment sizes ranged from 276–617 bp for trnH-psbA and 133–846 bp for matK genes.

**Table 2:** Variable success rates of two gene targets in amplifying and sequencing plant DNA in the crop of different mosquito species.

Mosquito species	N	Amplified (Sequenced)	
		trnH-psbA (total (success))	matK (total (success))
*Aedes aegypti*	42	8 (6)	0 (0)
*Aedes mcintoshi*	38	2 (2)	2 (0)
*Aedes ochraceus*	32	11 (3)	9 (0)
*Anopheles gambiae* s.l.	35	18 (15)	3 (3)
**Overall**	**147**	**39 (26)**	**14 (3)**

N = number of mosquitoes from which plant DNA were extracted.

**Fig 1:**
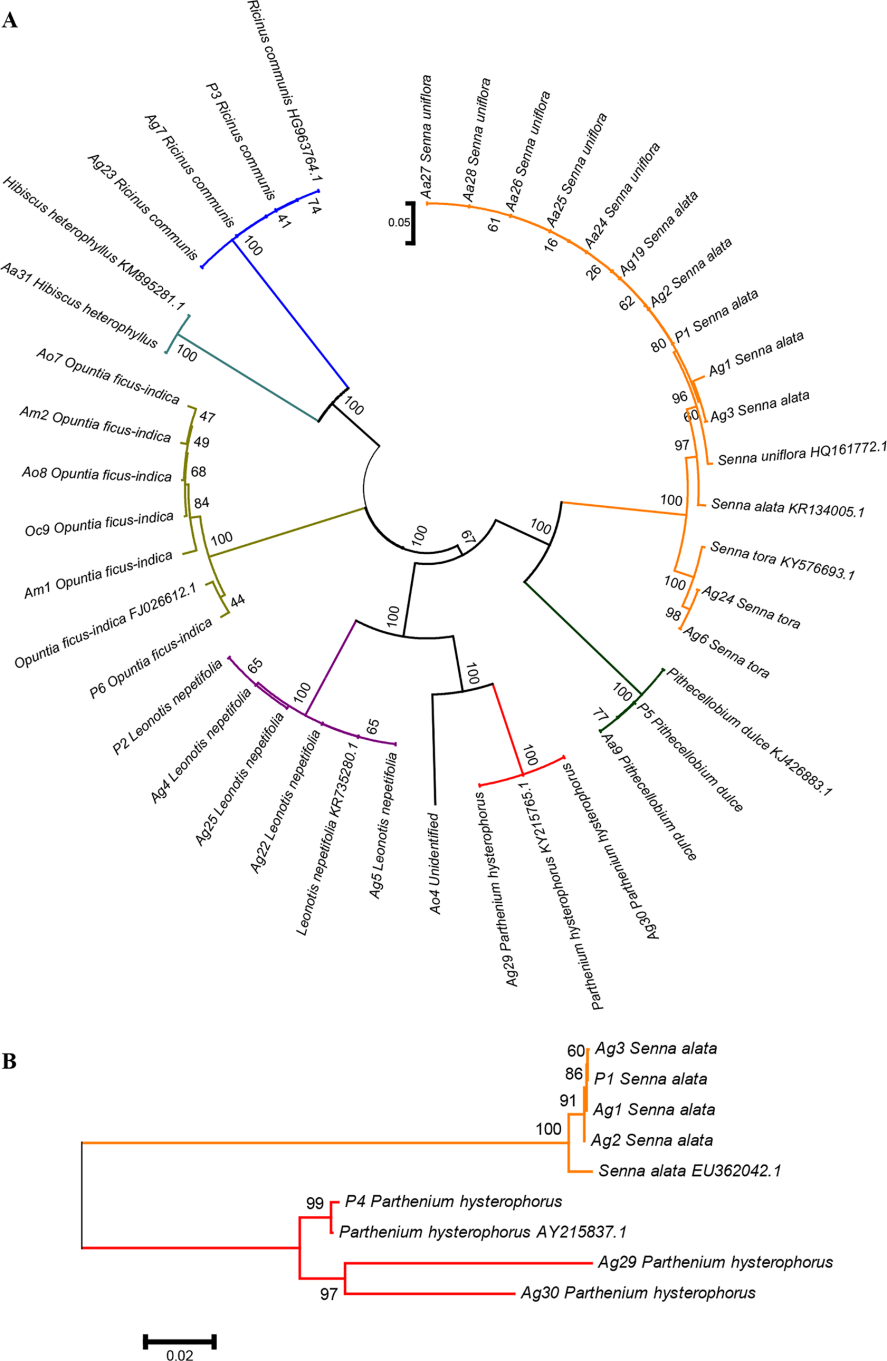
NJ phylogenetic trees from two gene targets showing plant species identified as natural host plants of the Afro-tropical mosquito species. A) Plant species identified using trnH-psbA gene targets as host plants for *Aedes aegypti*, *Aedes mcintoshi*, *Aedes ochraceus* and *Anopheles gambiae*. B) Plant species identified using matK gene targets as host plants for *Anopheles gambiae*. Plant species names with prefix Aa from *Aedes aegypti*, Am from *Aedes mcintoshi*, Ao from *Aedes ochraceus* and Ag represent those that were identified from *Anopheles gambiae*, the numbers being sample ID. Plant species with prefix P1-4 represent the plant samples sequences to confirm the identity of the mosquito host plants while those with suffixes are outgroups from GenBank with extension being accession numbers.

Blast searches of the sequences for each target in GenBank and further phylogeny showed strong support (bootstrap values 95% and above) and identified host plants as *Pithecellobium dulce* (Fabaceae), *Senna uniflora* (Fabaceae) and *Hibiscus heterophyllus* (Malvaceae) for *Ae*. *aegypti* (Fig 1A); *Opuntia ficus-indica* (Cactaceae) for *Ae*. *mcintoshi*; and *O*. *ficus-indica* and an unidentified plant species for *Ae*. *ochraceus* (Fig 1A); and *Senna alata* (Fabaceae), *Senna tora* (Fabaceae), *Ricinus communis* (Euphorbiaceae), *Parthenium hysterophorus* (Asteraceae) and *Leonotis nepetifolia* (Lamiaceae) for *An*. *gambiae* (Fig 1A and 1B). The plant identities were further corroborated by on-site botanical identification to confirm their presence and inclusion of matched sequences of extracted DNA in the analyses. These and putative sequences from the mosquito gut clustered together with strong bootstrap support in the phylogenetic analysis (Fig 1A and 1B).

### Diverse volatile organic compounds characterize the natural host plants of Afro-tropical mosquito species

**Fig 2:**
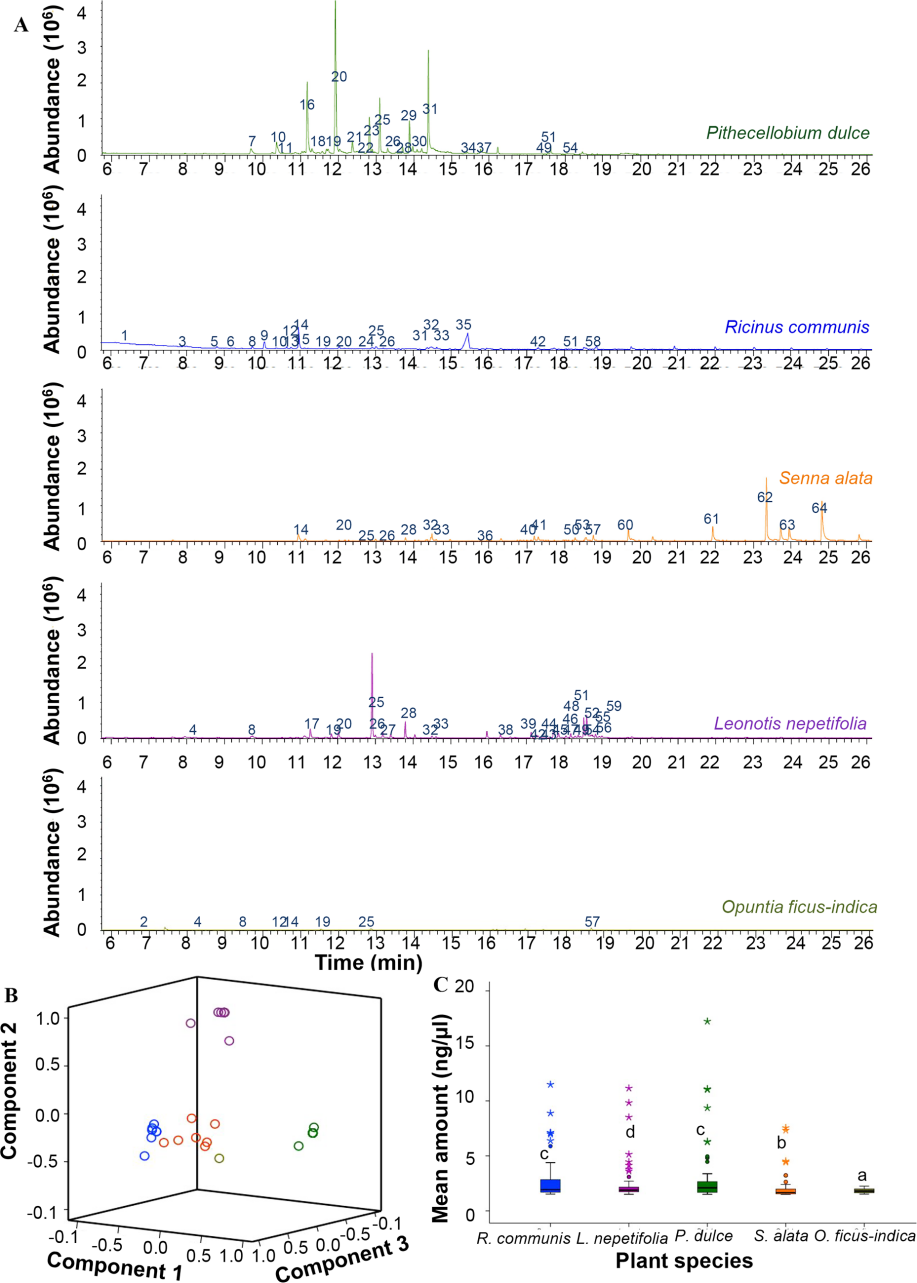
Variable chemical profiles of plant species used by different mosquito species as host plants. A) Representative profiles of headspace volatile organic compounds (VOCs) of different plant species as measured by coupled gas chromatography/mass spectrometry. The identities of the compounds labeled 1–73 representing VOCs from the five plant species, their retention times and Kovats indices are listed in S1 Table (additional information). B) Three-dimensional graphical representation of PCA which resolves the volatile profiles of the five plant species into three distinct clusters. PCA1 = 38%, PCA2 = 32% and PCA3 = 22%. C) Mean amounts of VOCs from the five plant species. Bars capped with different letters are significantly different. Circles and asterisk above the box plots represent outliers. Quantitative differences in the VOCs content of the five plants were detected using Univariate analysis of variance and Tukey HSD.

We analyzed headspace volatiles from five of the identified natural host plants *viz P*. *dulce*, *O*. *ficus-indica*, *L*. *nepetifolia*, *S*. *alata* and *R*. *communis*. The VOCs of the five different plant species were differentiated by unique chemical constituents of varying abundance (Fig 2A). Principal Component Analysis (PCA) resolved these chemical constituents into three clusters which accounted for more than 90% of the total variation (Fig 2B). PC1 explained 38% of the variation; PC2 explained 32% while PC3 explained 22% of the variation. PC1 was weighed positively by monoterpenoids and benzenoids, predominantly unique to *P*. *dulce*, while PC2 was positively contributed to by sesquiterpenes which were characteristically abundant in *L*. *nepotifolia* (S1 Table). PC3 was positively characterized by monoterpenes which were the key constituents detected in the VOCs of *R*. *communis* (S1 Table). The headspace volatile constituents of *S*. *alata* and *O*. *ficus-indica* contained benzenoids (S1 Table). (*E*)-β-Ocimene was present in the VOCs of all the five host plant species, while hexanal, (*E*)-2-hexen-1-ol, β-myrcene, benzaldehyde, α-pinene, nonanal, linalool oxide, decanal, methyl salicylate, (*E*)-β-caryophyllene and germacrene D were variably present in the volatiles of two or more plant species (S1 Table). Multivariate analysis of variance revealed significant quantitative differences in the volatile profiles of the five plants (F_(4, 1095)_ = 142.907, *P* < 0.001; Fig 2C).

### Afro-tropical mosquito species detect unique volatile organic compounds from their natural host plants

**Fig 3:**
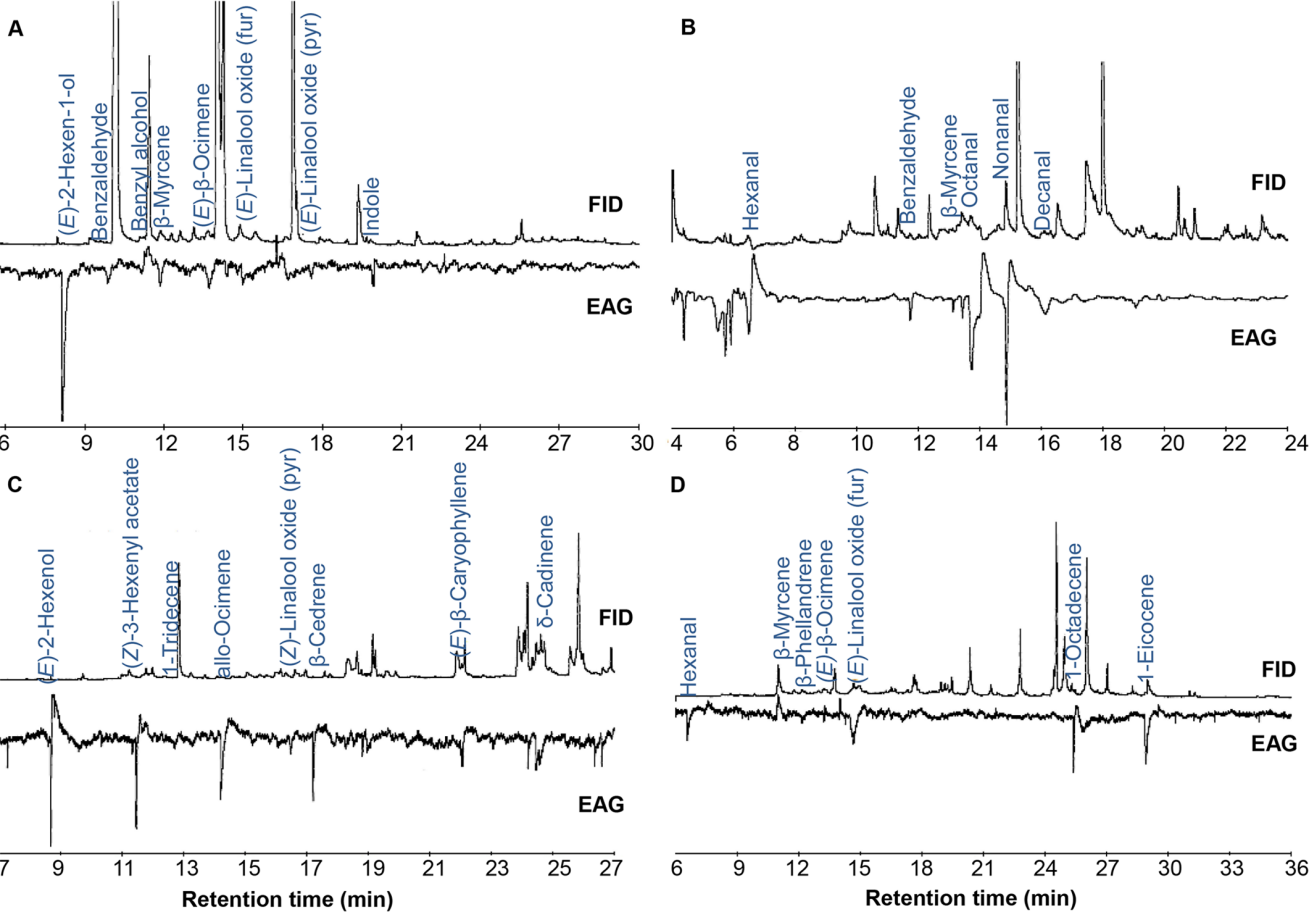
GC/EAD profiles of headspace volatiles collected from natural host plants of specific Afro-tropical mosquitoes. A) *Aedes aegypti gambiae* antennal detection of specific VOCs from *Pithecellobium dulce*.B) *Aedes mcintoshi* antennal detection of VOCs from *Opuntia ficus-indica*. C) *Anopheles gambiae* antennal detection of specific VOCs from *Leonotis nepetifolia*. D) *An*. *gambiae* antennal detection of specific VOCs from *Senna alata*.

To test if Afro-tropical mosquito species detect odors of their natural host plants, we employed coupled gas chromatography/electroantennographic detection (GC/EAD) and GC/mass spectrometry to isolate and identify VOCs that are detected by antennae of *Ae*. *aegypti*, *Ae*. *mcintoshi* and *An*. *gambiae*. Our assays revealed that the antennae of the three different mosquito species detected a total of 21 different VOCs, some of which were unique to their preferred host plants while others were common across two or more of the plant species. Antennae of *Ae*. *aegypti* detected 8 components in *P*. *dulce* headspace volatiles (Fig 3A), with 12 components detected by *Ae*. *mcintoshi* from *O*. *ficus-indica* (Fig 3B) while those of *An*. *gambiae* s.l. detected 13 and 7 components in the volatiles of *L*. *nepetifolia* and *S*. *alata*, respectively (Fig 3C and 3D). β-Myrcene and ocimene were detected by antennae of all the three different mosquito species from their respective host plants while hexanal, (*E*)-2-hexenol, and linalool oxide isomers, and benzaldehyde were variably detected by the three different mosquito species. On the other hand, antennae of the three different mosquito species also detected unique compounds from their respective host plants which included benzenoids (benzyl alcohol and indole) by *Ae*. *aegypti*, aldehydes (octanal, nonanal and decanal) by *Ae*. *mcintoshi*, and sesquiterpenes (β-cedrene, (*E*)-β-caryophyllene, α-humulene and δ-cadinene) and C-13, C-18 and C-20 alkenes by *An*. *gambiae* (Fig 3A, 3B, 3C and 3D).

**Fig 4:**
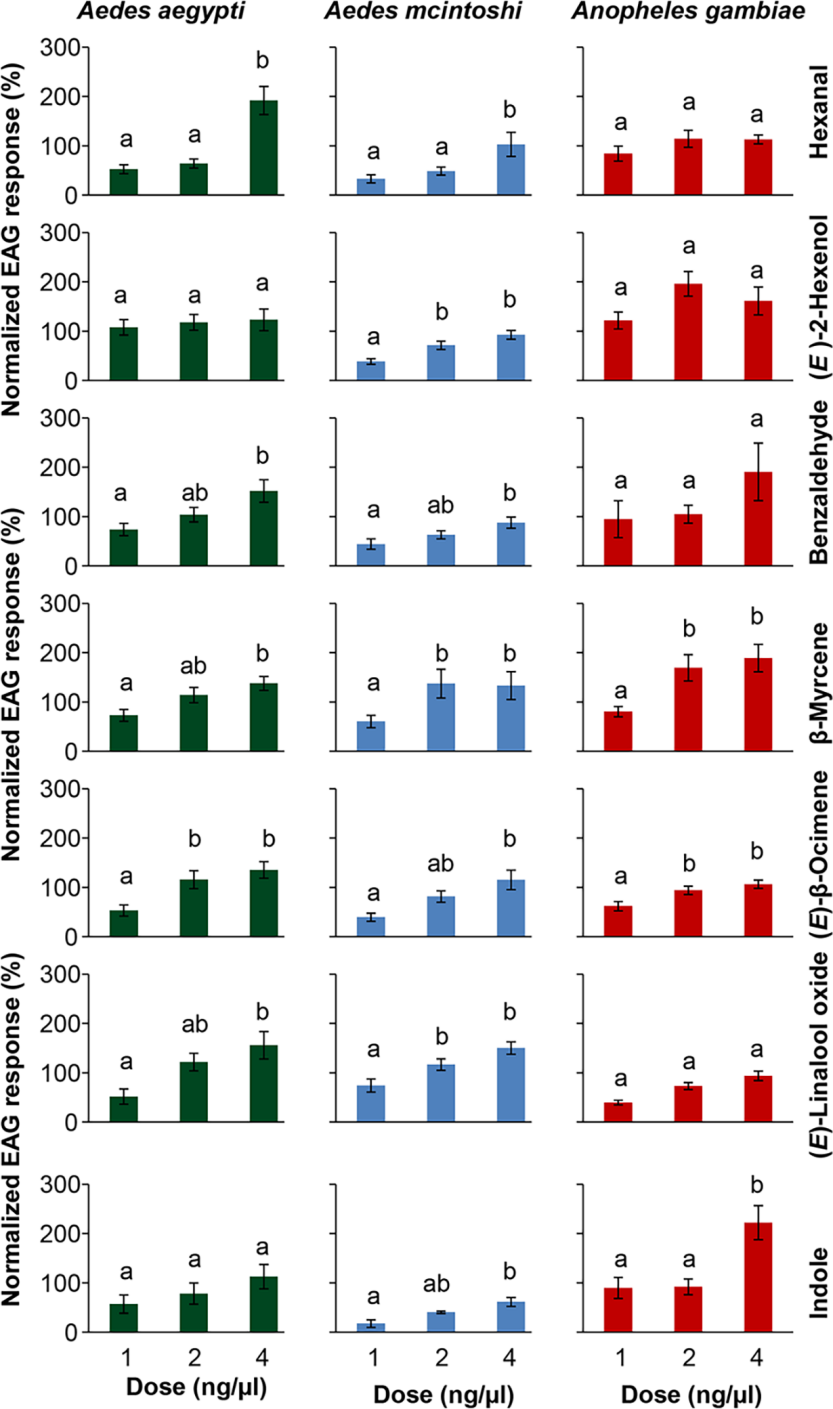
Electroantennographic detection responses of three Afro-tropical mosquito species to different doses of commonly detected plant volatile organic compounds. Variability in EAG responses were corrected by subtracting the responses to blanks (5 μl dichloromethane and bioassay filter paper) from each sample and the antennal response values normalized to a standard stimulus set at 100% (2 ng/μl 1-octen-3-ol). Bars capped with different letters are significantly different between the three doses. The differences in dose response were detected using ANOVA followed by Tukey post hoc test.

We then selected compounds which were detected by two or more mosquito species for further electrophysiological assays to confirm and compare their bioactivity. These included hexanal, (*E*)-2-hexen-1-ol, benzaldehyde, β-myrcene, (*E*)-β-ocimene and (*E*)-linalool oxide (the mass spectra of these six compounds are provided in S1A–S1G Fig). In addition, following isolation of indole as an EAG-active VOC from *Ae*. *aegypti* host plant and its known role as an oviposition cue for different mosquito species [51], we also included it in these electrophysiological assays. 1-octen-3-ol was used as reference compound. Antennal responses of the two *Aedes* species *Ae*. *mcintoshi* and *Ae*. *aegypti* to the seven compounds tested were dose dependent, while that of *An*. *gambiae* was dose-dependent to three of the compounds including β-myrcene, (*E*)-β-ocimene and indole (Fig 4).

**Fig 5:**
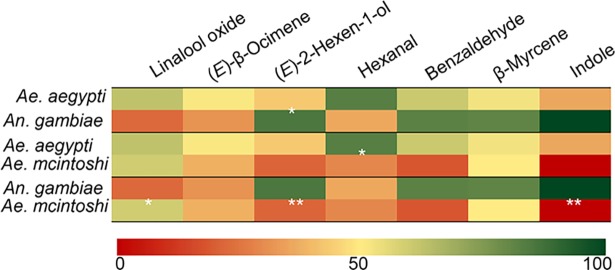
Heat map showing varying intensities of antennal responses to synthetic standards of identified compounds in three mosquito species. The heat maps are based on doses (4ng/μl) of each compound eliciting the highest antennal responses in the respective mosquito species. Green represent higher responses while red indicate lower responses. White asterisks denote significant differences between two mosquito species. Differences in antennal responses were detected using ANOVA and the means separated with Tukey post hoc test.

β-Myrcene and (*E*)-β-ocimene elicited consistent dose-dependent antennal response across the three different mosquito species. In depth analysis revealed significant differences in odor detection intensities between the three mosquito species (F_(2, 163)_ = 6.492, *P* < 0.01), with linalool oxide, (*E*)-2-hexenol, hexanal and indole variably detected by the three mosquito species (Fig 5).

## Discussion

Our findings confirm that plant feeding is common among the four Afro-tropical mosquito vectors of dengue, RVF and malaria among other diseases, evidenced by a significant proportion of anthrone-positive mosquitoes. For the first time, we also identify the host plants fed upon in nature from the habitats of these vectors using DNA barcoding. Plants identified included *P*. *dulce*, *S*. *uniflora* and *H*. *heterophyllus* for *Ae*. *aegypti*, *O*. *ficus-indica* for *Ae*. *mcintoshi* and *Ae*. *ochraceus*, and *L*. *nepetifolia*, *S*. *alata* and *S*. *tora* for *An*. *gambiae*. This study represents the first evidence of plant feeding among RVF vectors. Also, some of the plant species which had been presumed to be potential host plants for malaria vectors due to their presence near human dwellings in malaria endemic regions were confirmed as host plants. These included the highly aggressive invasive plants *P*. *hysterophorus* and *R*. *communis* [27, 28]. The implications of these findings in the context of control of mosquito-borne diseases include: 1) the precision of attractive toxic sugar baits can be greatly improved by application of the baits on preferred natural host plants as opposed to random selection of plants to be laced with insecticides, 2) some of these plants might have metabolites that impact on pathogen-vector interactions which can be exploited for development of chemotherapeutics and transmission blocking agents, and 3) chemical cues utilized by these mosquito species in locating their preferred natural host plants can be harnessed for development of odor-bait technology to be used in vector surveillance and control.

Previous studies have suggested that mosquitoes mainly feed on plant nectars, extrafloral nectaries and honeydew [41], with limited evidence of tissue feeding [19]. The isolation of plant DNA from field collected mosquitoes points to plant tissue feeding in addition to nectars. Plant nectars are mainly composed of sugars, with some plants having small amounts of amino acids and proteins [52, 53], and trace amounts of DNA [54]. Our results show discrepancy in the number of fructose positive mosquitoes and those from which plant DNA was successfully isolated, with only about 25% and 8% of fructose positive mosquitoes amplified for trnH-psbA and matK gene targets, respectively. Junilla et al. [35] attributed the detection of plant DNA in mosquitoes fed on flowering plants to possible presence of DNA in the nectar or plant tissue feeding. We have observed in our laboratory plant feeding assays that mosquitoes feed not only on plant nectar but also pierce through the stems and leaf stalks (data not included). Similar observations were made by Junilla et al. [35]. These observations strongly suggest that mosquitoes possibly pierce through plant tissue to draw nutrients from plant sap in addition to nectars. In such a scenario, the mosquitoes would be predisposed to a few plant metabolites which may impact on their fitness and pathogen transmission potential, as evidenced in previous studies [21, 25, 26]. Given that these Afro-tropical mosquito species can discriminate their host plants from a plethora of plant species present in their habitats, with more than one host plant identified for three of the mosquito species, it is possible that the mosquitoes forage on different host plants for different fitness-related benefits. This, however, does not rule out the possibility of chance feeding depending on the seasonal availability of a given plant species, an aspect that warrants further research.

The present study further documents both qualitative and quantitative differences in the VOCs of five of the identified host plants. The variable headspace volatile profiles are not surprising; similar observations even within the same plant species from different cultivars, seasons and geographical locations has been made before [55–57]. Besides, it is probable that these plants utilize different metabolic pathways to give them the unique fragrance necessary for a competitive advantage in the event of scarcity of certain shared resources such as pollinators, parasitoids and self-defense [58]. Olfactory cues play a central role in herbivorous insect- host plant interactions, as evidenced by previous studies [40, 59, 60]. In a complex environment permeated with many odor plumes from different plant species, plant feeding insects are expected to evolve mechanisms that allow them to discriminate biologically relevant chemical cues for resources that confer fitness [60]. Consequently, the finding that mosquitoes can discriminate beneficial host plants from a plethora of plant species in their habitats with variable VOCs is intriguing. Noteworthy, however, is the fact that some of the VOCs such as (*E*)-β-ocimene, β-myrcene, hexanal, (*E*)-2-hexen-1-ol, benzaldehyde, α-pinene, nonanal, linalool oxide, decanal, methyl salicylate, (*E*)-caryophyllene and germacrene D were common to more than half of the plants analyzed, albeit in variable amounts. These compounds have been implicated in plant-insect interactions either as pollinator attractants or in plant defense to attract natural enemies of detrimental herbivores [61, 62]. In addition, some of these compounds have also been shown to be utilized by disease vectors to locate either vertebrate host [37, 44] or host plant [40, 41].

The electrophysiological assays revealed a range of specific compounds from different host plants which elicited antennal activity. Among VOCs that were common across two or more plant species, β-myrcene, hexanal, (*E*)-2-hexen-1-ol, benzaldehyde and the different isomers of ocimene and linalool oxide were detected by two or more mosquito species from the VOCs of their respective host plants. On the other hand, the three different mosquito species also detected unique classes of VOCs from their host plants. These included benzenoids by *Ae*. *aegypti*, aldeydes and a benzenoid by *Ae*. *mcintoshi*, and sesquiterpenes and alkenes by *An*. *gambiae*. These results point to the adaptive nature of plant odor reception by different mosquito species that allows them to discriminate beneficial from non-beneficial plants. (*E*)-Linalool oxide and (*E*)-β-ocimene have been shown to be among the VOCs from different plant species that elicit antennal activity and behavioral responses in *Ae*. *aegypti*, *An*. *gambiae* and *Culex pipiens* [40, 43, 56, 63]. On the other hand, linalool oxide and benzaldehyde have also been reported as components of human odors that elicit antennal activity in *An*. *gambiae* [64] and *Ae*. *aegypti* [65], respectively. Interestingly, indole which has been reported as an oviposition site attractant for different *Culex* and *Aedes* species [51], was antennally detected by *Ae*. *aegypi* from the VOCs of its host plant *P*. *dulce*. Indole has been shown to be a by-product of both bacterial degradation of tryptophan [66] and plants [67]. Taken together, these overlaps in odor detection by different mosquito species across different host plants, with some of the compounds having been identified from vertebrate hosts and oviposition sites, point to a conserved nature of receptors for certain biologically relevant chemical cues.

To further assess the potential differences in specificities of plant odor detection between the three different mosquito species, we conducted dose-dependent electrophysiological assays using seven of the identified VOCs. The seven compounds included hexanal, (*E*)-2-hexen-1-ol, benzaldehyde, β-myrcene, (*E*)-β-ocimene, (*E*)-linalool oxide and indole. Our results showed variable dose response detection to the seven compounds by the three different mosquito species in electrophysiological assays, with β-myrcene and (*E*)-β-ocimene eliciting significant dose response across all the three species. There were no differences in the sensitivity of the three different mosquito species to β-myrcene and (*E*)-β-ocimene, possibly pointing to the conserved nature of the receptors for these two compounds across different mosquito species. Thus, it is possible that mosquitoes use both β-myrcene and (*E*)-β-ocimene to detect the presence of potential host plants, with the other volatiles playing a background role to the overall chemical signature in determining the suitability of the plant as a potential nutrient source. Molecules of high biological significance have been suggested to be encoded by narrowly tuned odor receptors [68], which have been shown to be highly conserved both quantitatively and qualitatively across different mosquito species [51]. While considerable effort has been dedicated towards structural elucidation of vertebrate [42, 64] and oviposition [51] odor receptors in different mosquito species, plant odor receptors in these disease vectors is yet to be fully explored. Our results point to a similar odor partitioning in mosquito-plant interactions as is the case in vertebrate host and oviposition site location. While this study presents a significant step in elucidating important plant VOCs mediating mosquito-host plant interactions, additional studies to identify plant odor receptors are necessary to understand their nature and help narrow down on key plant VOCs that can be used in their management. In addition, further studies are needed to elucidate the role these compounds and other identified VOCs play in the behavior of the different mosquito species.

Overall, this study presents a significant milestone in the quest for novel control strategies to either supplement or replace existing ones. For both dengue and RVF, no viable vaccine or treatment exists, at least for the human cases [10]. Consequently, vector control constitutes a key pillar in their eradication/containment efforts. Besides, both diseases are characterized by cyclic patterns of outbreaks with low viral activity during the inter-epidemic periods [2, 3, 6, 38]. Thus, accurate and efficient monitoring tools are needed to predict outbreaks, a task which can be greatly complemented by plant-based odors identified in this study. Similarly, new control tools incorporating vector ecology are needed to sustain the achievements recorded in reducing malaria incidence and further move towards elimination [10].

In conclusion, this study demonstrates that the Afro-tropical mosquito species feed on various plant species available within their ecological ranges. Interestingly, they use specific chemical cues to interact with their natural host plants, some of which are common to all the three different mosquito species while others are species-specific. These findings provide a critical insight into chemical communication that underpins mosquito-plant interactions and present a unique opportunity for advancement of plant-based mosquito control strategies. We, however, take note of the fact that the plants identified in this study might not be entirely representative of the full spectra of plants fed upon by these mosquito species in their respective ecology as these are likely to vary with season. In addition, the low success rates of the two gene targets used in this study is indicative of the likelihood that some plant species might not have been detected, hence the need for further screening using additional gene targets. Nonetheless, these findings provide new insights critical in understanding the ecological drivers of the emergence of vector-borne tropical diseases and a baseline for new control strategies.

## Supporting information

S1 Table List of compounds identified from five host plants of Afro-tropical mosquito species and relative amounts ± SEM (ng). The compounds were identified from headspace volatiles of LN = *Lenonotis nepetifolia*, RC = *Ricinus communis*, SA = *Senna alata* (host plants of *Anopheles gambiae*), PD = *Pithecellobium dulce* (host plant of *Aedes aegypti*) and OFI = *Opuntia ficus-indica* (host plant of *Aedes mcintoshi* and *Aedes ochraceus*).
(DOCX)

S1 Fig Mass spectra and chemical structures of electrophysiologically active compounds which were confirmed with synthetic standards. A) hexanal, B) (*E*)-2-hexenol, C) β-myrcene, D) (*E*)-β-ocimene, E) (*Z*)-linalool oxide (furanoid), F) (*Z*)-linalool oxide (pyranoid), G) indole, and H) benzaldehyde.
(TIF)
