# Supplementary material for: Host plant forensics and olfactory-based detection in Afro-tropical mosquito disease vectors
Source: PLoS Negl Trop Dis. 2018 Feb 20;12(2):e0006185. doi: 10.1371/journal.pntd.0006185 (PMC5834208; doi:10.1371/journal.pntd.0006185)
Supplement: S1 Table — The compounds were identified from headspace volatiles of LN = Lenonotis nepetifolia, RC = Ricinus communis, SA = Senna alata (host plants of Anopheles gambiae), PD = Pithecellobium dulce (host plant of Aedes aegypti) and OFI = Opuntia ficus-indica (host plant of Aedes mcintoshi and Aedes ochraceus). (DOCX) [file pntd.0006185.s001.docx]

Table S1 List of compounds identified from five host plants of Afro-tropical mosquito species and relative amounts ± SEM (ng).

|  | **Compound** | **RT** | **RI** | **%** | **Class** | **LN** | **PD** | **RC** | **SA** | **OFI** |
| --- | --- | --- | --- | --- | --- | --- | --- | --- | --- | --- |
| 1 | 1-Octene | 6.11 | 776 | 95 | Alkene | - | - | 2.98±0.10 | - | - |
| 2 | Hexanal^ǂ^ | 6.87 | 804 | 72 | Aldehyde | - | - | - | 1.50±0.00 | 1.63±0.10 |
| 3 | (*Z*)-3-Hexen-1-ol^ǂ^ | 8.01 | 848 | 93 | Alcohol | 1.70±0.17 | - | - | - | - |
| 4 | (*E*)-2-Hexen-1-ol^ǂ^ | 8.57 | 870 | 81 | Alcohol | 1.73±0.15 | 1.58±0.07 | 1.95±0.20 | - | - |
| 5 | 1-Decene | 8.82 | 879 | 72 | Alkene | 1.55±0.01 | - | 1.79±0.12 | - | - |
| 6 | Methyl (3*E*)-hexenoate | 9.70 | 917 | 96 | Ester | - | 2.37±0.14 | - | - | - |
| 7 | α-Pinene^ǂ^ | 9.76 | 918 | 91 | Monoterpene | 1.76±0.11 | - | 2.46±0.77 | - | 1.97±0.28 |
| 8 | Camphene | 10.08 | 933 | 98 | Monoterpene | 1.70±0.08 | - | 3.75±1.70 | - | - |
| 9 | Benzaldehyde^ǂ^ | 10.23 | 940 | 95 | Benzenoid | - | 1.68±0.12 | 2.07±0.41 | - | 1.55±0.04 |
| 10 | Methyl (2*E*)-hexenoate | 10.37 | 946 | 96 | Ester | - | 3.02±0.36 | - | - | - |
| 11 | β-Pinene^ǂ^ | 10.66 | 959 | 91 | Monoterpene | - | - | 2.56±0.89 | - | 1.54±0.01 |
| 12 | 1-Octen-3-ol^ǂ^ | 10.74 | 962 | 76 | Alcohol | - | - | 1.96±0.24 | - | - |
| 13 | β-Myrcene^ǂ^ | 10.91 | 970 | 94 | Monoterpene | - | 2.16±0.29 | 6.96±2.37 | 1.96±0.44 | 1.79±0.00 |
| 14 | Decane | 11.04 | 976 | 94 | Alkane | - | - | 2.19±0.50 | - | - |
| 15 | Octanal^ǂ^ | 11.13 | 980 | 87 | Aldehyde | - | 1.74±0.14 | 1.66±0.08 | - | 1.71±0.14 |
| 16 | (*Z*)-3-Hexenyl acetate^ǂ^ | 11.28 | 987 | 90 | Ester | 2.15±0.29 | 6.31±3.05 | - | - | - |
| 17 | Limonene^ǂ^ | 11.68 | 1006 | 94 | Monoterpene | - | 1.83±0.15 | 2.04±0.38 | - | 1.70±0.10 |
| 18 | β-Phellandrene | 11.68 | 1006 | 74 | Monoterpene | - | - | - | 1.69±0.04 | - |
| 19 | (*Z*)-β-Ocimene^ǂ^ | 11.84 | 1016 | 96 | Monoterpene | 1.97±0.12 | 1.89±0.33 | - | - | - |
| 20 | (*E*)-β-Ocimene^ǂ^ | 12.03 | 1028 | 97 | Monoterpene | 2.35±0.19 | 11.09±6.14 | 2.51±0.67 | 1.57±0.00 | 2.06±0.53 |
| 21 | (*Z*)-Linalool oxide (furanoid) ^ǂ^ | 12.43 | 1052 | 91 | Monoterpene | - | 2.87±0.25 | - | - | - |
| 22 | Methyl benzoate | 12.77 | 1072 | 74 | Benzenoid | - | 1.73±0.21 | - | - | - |
| 23 | Linalool^ǂ^ | 12.84 | 1077 | 90 | Monoterpene | - | 3.25±1.56 | - | - | - |
| 24 | Undecane | 12.92 | 1081 | 94 | Alkane | 9.83±1.32 | - | 1.85±0.03 | 1.56±0.05 | - |
| 25 | Nonanal^ǂ^ | 13.00 | 1086 | 91 | Aldehyde | 1.96±0.05 | 1.89±0.21 | 2.24±0.27 | 1.61±0.08 | 1.86±0.08 |
| 26 | (*E*)-Linalool oxide (furanoid) ^ǂ^ | 13.20 | 1098 | 87 | Monoterpene | 1.90±0.37 | 10.89±2.46 | 1.76±0.16 | 1.57±0.05 | - |
| 27 | allo-Ocimene | 13.31 | 1100 | 97 | Monoterpene | 2.05±0.17 | 2.12±0.54 | - | - | - |
| 28 | Benzyl acetate | 13.90 | 1139 | 96 | Benzenoid | - | 3.01±1.45 | - | - | - |
| 29 | (*Z*)-Linalool oxide (pyranoid) ^ǂ^ | 14.01 | 1151 | 90 | Monoterpene | 1.65±0.14 | 2.27±0.08 | - | - | - |
| 30 | (*Z*)-3-Hexenyl butanoate | 14.21 | 1158 | 78 | Ester | - | 2.01±0.41 | - | - | - |
| 31 | Methyl salicylate^ǂ^ | 14.47 | 1173 | 93 | Benzenoid | 1.68±0.01 | 6.27±4.76 | - | 1.87±0.33 | - |
| 32 | Octanoic acid | 14.52 | 1176 | 60 | Carboxylic acid | - | - | 5.40±1.64 | - | - |
| 33 | Decanal^ǂ^ | 14.64 | 1183 | 87 | Aldehyde | 1.63±0.02 | - | 2.00±0.12 | 1.68±0.03 | 1.78±0.22 |
| 34 | *p*-Ethyl acetophenone | 15.35 | 1228 | 96 | Benzenoid | - | 1.70±0.18 | - | - | - |
| 35 | Thymol | 15.76 | 1255 | 95 | Benzenoid | - | 2.23±0.06 | - | - | - |
| 36 | Tridecane | 15.95 | 1268 | 97 | Alkane | 2.16±0.13 | - | - | 1.56±0.03 | - |
| 37 | Indole^ǂ^ | 15.85 | 1261 | 94 | Benzenoid | - | 3.89±0.02 | - | - | - |
| 38 | α-Cubebene | 16.75 | 1323 | 81 | Sesquiterpene | 2.13±0.21 | - | - | - | - |
| 39 | 1-Tetradecene | 17.12 | 1350 | 72 | Alkene | - | - | - | 1.75±0.24 | - |
| 40 | β-Bourbonene | 17.23 | 1358 | 90 | Sesquiterpene | 1.82±0.09 | - | - | - | - |
| 41 | Tetradecane | 17.26 | 1360 | 87 | Alkane | - | - | 1.90±0.13 | 1.76±0.18 | - |
| 42 | β-Gurjunene | 17.29 | 1362 | 93 | Sesquiterpene | 1.83±0.01 | - | - | - | - |
| 43 | α-Cedrene^ǂ^ | 17.30 | 1363 | 90 | Sesquiterpene | 1.55±0.04 | 1.59±0.03 | - | - | - |
| 44 | α-Copaene | 17.65 | 1388 | 90 | Sesquiterpene | 1.92±0.38 | - | - | - | - |
| 45 | (*E*)-β-Caryophyllene^ǂ^ | 17.73 | 1394 | 99 | Sesquiterpene | 2.67±0.04 | 2.15±0.22 | 2.17±0.35 | - | - |
| 46 | β-Copaene | 17.85 | 1402 | 96 | Sesquiterpene | 2.16±0.14 | - | - | - | - |
| 47 | β-Cubebene | 18.05 | 1418 | 93 | Sesquiterpene | 1.81±0.07 | - | - | - | - |
| 48 | α-Humulene | 18.17 | 1427 | 96 | Sesquiterpene | 2.09±0.16 | 1.59±0.03 | - | - | - |
| 49 | Dauca-5,8-diene | 18.28 | 1435 | 94 | Sesquiterpene | 1.72±0.04 | - | - | - | - |
| 50 | Unidentified | 18.31 | 1438 | 72 | Sesquiterpene | - | - | - | 1.69±0.18 | - |
| 51 | Longifolene | 18.37 | 1442 | 93 | Sesquiterpene | 1.73±0.05 | - | - | - | - |
| 52 | Germacrene D^ǂ^ | 18.51 | 1453 | 98 | Sesquiterpene | 3.61±0.52 | - | 2.91±0.75 | - | - |
| 53 | Pentadecane | 18.58 | 1459 | 93 | Alkane | - | - | - | 1.85±0.17 | - |
| 54 | Premnaspirodiene | 18.59 | 1459 | 97 | Sesquiterpene | 4.46±0.68 | - | - | - | - |
| 55 | λ-Gurjunene | 18.71 | 1469 | 91 | Sesquiterpene | 2.10±0.06 | - | - | - | - |
| 56 | 2,4-Di-tert-butylphenol | 18.77 | 1473 | 93 | Benzenoid | - | - | - | 1.84±0.32 | - |
| 57 | Unidentified | 18.77 | 1473 | 72 | - | - | - | - | - | 1.61±0.00 |
| 58 | δ-Cadinene | 18.93 | 1486 | 64 | Sesquiterpene | 1.74±0.01 | - | - | - | - |
| 59 | δ-Amophene | 19.01 | 1492 | 94 | Sesquiterpene | 1.81±0.01 | - | - | - | - |
| 60 | (*Z*)-7-Hexadecene | 19.70 | 1548 | 98 | Alkene | - | - | - | 2.07±0.56 | - |
| 61 | *(E)-*15-Heptadecanal | 21.94 | 1756 | 99 | Aldehyde | - | - | - | 2.38±0.85 | - |
| 62 | Unidentified | 23.34 | 1897 | 99 | Sesquiterpene | - | - | - | 4.53±3.03 | - |
| 63 | Unidentified | 23.97 | 1965 | 96 | Alkane | - | - | - | 2.38±0.85 | - |
| 64 | 1-Octadecene | 24.80 | 2054 | 97 | Alkene | - | - | - | 4.43±2.90 | - |

The compounds were identified from headspace volatiles of LN = *Lenonotis nepetifolia*, RC = *Ricinus communis*, SA = *Senna alata* (host plants of *Anopheles gambiae*), PD = *Pithecellobium dulce* (host plant of *Aedes aegypti*) and OFI = *Opuntia ficus-indica* (host plant of *Aedes mcintoshi* and *Aedes ochraceus*). RT = retention time, RI = Kovats Retention Index, ǂ denotes compounds whose identities were confirmed with synthetic standards. Other compounds identified based on mass spectral library data (Adams2.L, Chemecol.L and NIST05a.L).
