# Supplementary figures and images for: Host plant forensics and olfactory-based detection in Afro-tropical mosquito disease vectors
Source: PLoS Negl Trop Dis. 2018 Feb 20;12(2):e0006185. doi: 10.1371/journal.pntd.0006185 (PMC5834208; doi:10.1371/journal.pntd.0006185)

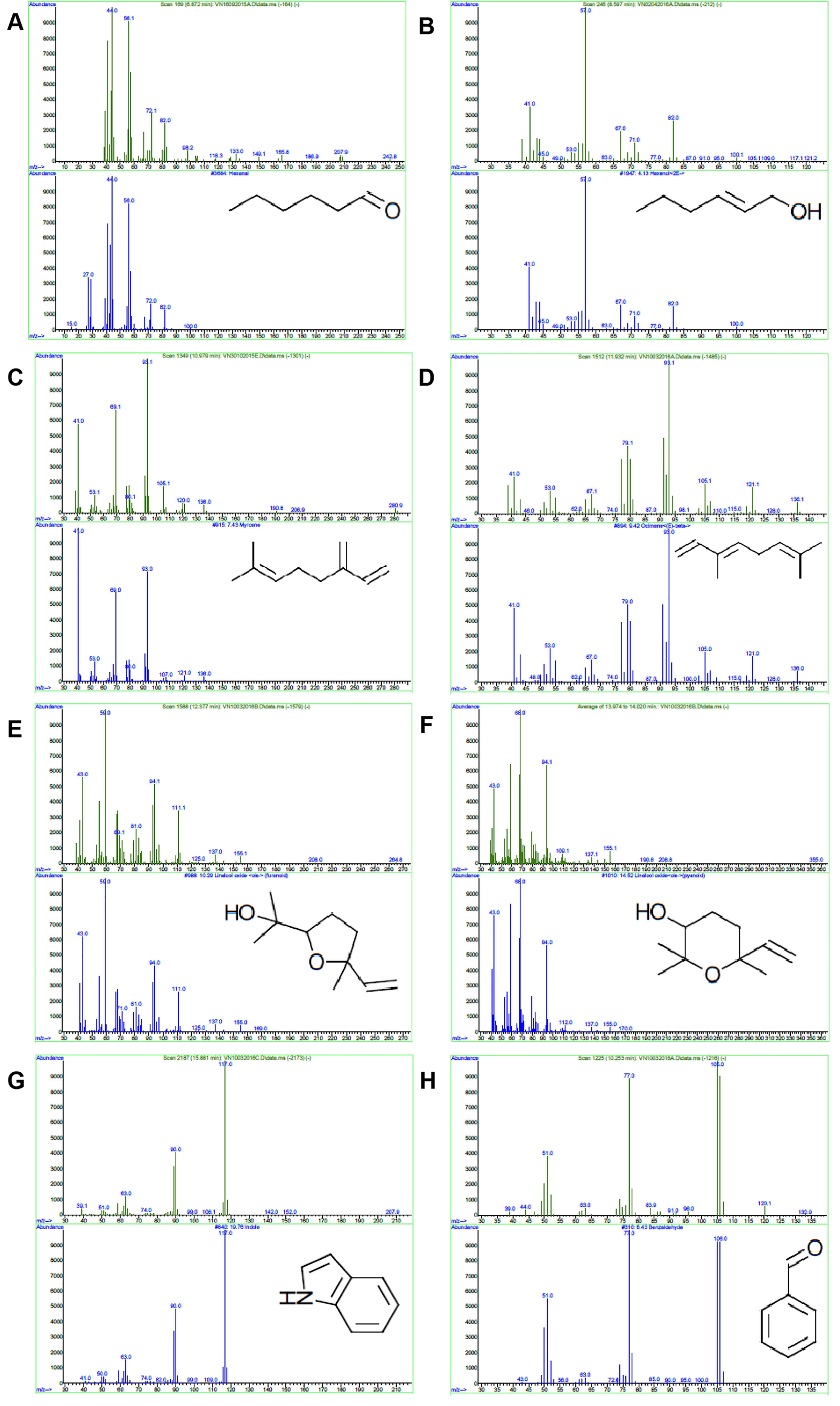

Supplement: S1 Fig — A) hexanal, B) (E)-2-hexenol, C) β-myrcene, D) (E)-β-ocimene, E) (Z)-linalool oxide (furanoid), F) (Z)-linalool oxide (pyranoid), G) indole, and H) benzaldehyde. (TIF) [file pntd.0006185.s002.tif]
